# Management of classical Hodgkin lymphoma: a look at up to date evidence and current treatment approaches

**DOI:** 10.1186/s40164-022-00360-4

**Published:** 2022-12-27

**Authors:** Walter Hanel, Alex F. Herrera, Narendranath Epperla

**Affiliations:** 1 Division of Hematology, Department of Medicine, The James Cancer Hospital and Solove Research Institute, The Ohio State University, 460 W 10th Ave, Columbus, OH 43210 USA; 2 Department of Hematology and Hematopoietic Cell Transplantation, City of Hope, 1500 E Duarte Rd, Duarte, CA 91010 USA; 3 The Ohio State University Comprehensive Cancer Center, 1110E Lincoln Tower, 1800 Cannon Drive, Columbus, OH 43210 USA

**Keywords:** Hodgkin lymphoma, cHL, Brentuximab vedotin, Nivolumab, Pembrolizumab

## Abstract

The treatment landscape of classical Hodgkin lymphoma (cHL) has undergone significant changes over the past 20 years. Gradual improvements have been made in the management of cHL patients, particularly in prolonging the survival rate for those in the relapsed setting. Most of these improvements came with the addition of brentuximab vedotin and PD1 blockade (nivolumab and pembrolizumab) into the current cHL treatment algorithms. On the other hand, the treatment approach to cHL has become more complex than ever before, with multiple ways to add and sequence therapies to achieve long-term remission. In this review, we will discuss the most up-to-date evidence on the management of cHL patients with the inclusion of ongoing clinical trials in cHL. We will provide a general overview of the current therapeutic landscape of cHL in light of these most recent data. We conclude with our perspective on how the approach to cHL treatment may evolve in the future.

## Introduction

Hodgkin lymphoma is a malignancy derived from B-lymphocytes recognized pathologically by its characteristic paucity of malignant cells and associated rich infiltrate of normal immune cells first described by Carl Sternberg and subsequently by Dorothy Reed in 1898 and 1902, respectively [1]. Nearly 95% of Hodgkin lymphoma is currently classified as classical Hodgkin lymphoma (cHL) while the remaining 5% is classified as nodular lymphocyte predominant Hodgkin’s lymphoma (NLPHL) under the current World Health Organization classification of lymphoid neoplasms [2]. The projected incidence of cHL in the US in 2022 is 8540 cases based on the Surveillance, Epidemiology and End Results Program. With current treatment approaches, the 5 year overall survival (OS) for patients with early stage (stages I–II) and advanced stage (stages III–IV) cHL is 92% and 82%, respectively [3].

Despite these excellent outcomes, around 920 people are estimated to die from cHL in the year 2022 [4], with mortality even higher in elderly patients. In addition, despite high cure rates with combined modality therapy or chemotherapy in the front-line setting, the risk of long-term side effects, including secondary malignancies, cardiovascular disease, cardiac dysfunction, and infertility are higher in patients treated with these traditional approaches compared to the general population (reviewed in [5]). Thus, the approach to achieve superior outcomes while minimizing toxicity for patients with cHL has remained the subject of intense investigation in the modern era. Over the past two decades, new treatment approaches, including the concept of risk-adapted treatment based on interim responses as well as novel biologic therapies, including the anti-CD30 antibody–drug conjugate (brentuximab vedotin, [BV]) and the anti-PD1 antibodies, have not only revolutionized the way we treat cHL but have also provided numerous new pathways of investigation on how to best incorporate these agents into treatment algorithms to achieve the optimal outcomes. In this article, we review the current standard of care for cHL, how novel agents are revolutionizing our approach to cHL, and how the standard of care may look in the near future. We will end by looking at important questions in the field currently and how these questions may be answered by ongoing and future research studies.

## Frontline treatment of cHL

### Early stage cHL

Doxorubicin, bleomycin, vinblastine, and dacarbazine (ABVD) still forms the backbone for the management of early-stage cHL patients in those who can tolerate anthracycline based chemotherapy. Historically, combined modality therapy (CMT, chemotherapy and radiation therapy [RT]) has been the mainstay of treatment for early-stage favorable cHL [6], however, PET adapted approaches have modified our management in these patients. The notable studies incorporating PET adapted approaches including the RAPID trial [7], CALGB/ALLIANCE 50604 study [8], the H10 EORTC/LYSA trial, and the German Hodgkin study group (GHSG) HD16 trial [9, 10] have shown acceptable outcomes while minimizing the use of RT. However, it is important to note that the omission of RT leads to inferior PFS in early-stage cHL compared to CMT.

While CMT remains the standard of care, BV and PD-1 blockade have both been studied in the frontline setting in patients with early-stage disease, particularly in patients with unfavorable disease, to improve outcomes while sparing bleomycin. In a randomized phase II trial, patients (*n* = 170) were randomized to receive either ABVD X 4 or BV + AVD X 4 cycles with both arms receiving 30 Gy of involved node RT as consolidation [11]. The primary endpoint was PET negativity after 2 cycles. The BV + AVD arm had 82.3% of patients with PET negativity compared to 75.4% in the ABVD arm, with 2 year PFS of 97.3% and 92.6%, respectively. In another pilot phase 2 study in early-stage unfavorable patients, BV was added to 4 cycles of AVD followed by randomization into 4 cohorts with a gradual reduction in radiation dosage: cohort 1 had 30 Gy of involved site RT (ISRT) (*n* = 30), cohort 2 had 20 Gy of ISRT (*n* = 29), cohort 3 had 30 Gy of consolidation volume radiotherapy (CVRT) (*n* = 29), and cohort 4 had no RT (*n* = 29) [12]. The CR rates in each cohort were 93%, 100%, 93%, and 97%, respectively, with 2 year PFS at 93%, 97%, 90%, and 97%. These results demonstrate excellent results with BV + AVD in the early stage unfavorable setting. Larger confirmatory studies to demonstrate superiority or non-inferiority of BV + AVD especially as an RT-sparing strategy in the early stage, unfavorable patients are warranted.

Pembrolizumab was investigated in a small phase II study of patients with early-stage unfavorable cHL (*n* = 30) in which pembrolizumab (200 mg) was initially given for 3 cycles every 3 weeks followed by AVD for 4–6 cycles [13, 14]. With pembrolizumab alone, 37% of patients were able to achieve a complete metabolic response (CMR). All patients went on to achieve a CMR after 2 cycles of AVD. The GHSG evaluated the use of nivolumab in a phase II study in the front-line setting for patients (*n* = 109) with unfavorable cHL either by a concurrent treatment approach (Nivo-AVD X 4 cycles) or a sequential approach (Nivo X 4 doses ->  Nivo-AVD X 2 -> AVD X 2). The CR rate was 90% and 94% with a 12 month PFS of 100% and 98% for concurrent and sequential therapies, respectively. These studies show the feasibility of incorporating immunotherapy into bleomycin sparing chemotherapy approaches in the frontline treatment of early-stage unfavorable patients with very good response rates.

In an ongoing PET adapted phase II study of early-stage cHL incorporating novel agents (NCT03712202) after receiving ABVD X 2, patients will be randomized either based on their Deauville score or baseline bulky disease status into either one of four arms: for Deauville 1–3 without bulky disease, patients will receive either (1) BV + nivolumab for 3 cycles, or (2) ABVD X 2 followed by nivolumab every 2 weeks for 3 months. Patients with bulky disease achieving a Deauville 1–3 will receive ABVD X 2 followed by nivolumab every 2 weeks for 3 months. Patients not achieving a Deauville 1–3 will receive BV + AVD X 4 followed by nivolumab every 2 weeks for 3 months.

### Advanced stage cHL

Given the relatively high response rates with ABVD therapy and the availability of effective salvage treatment options for advanced stage cHL, most of the research in frontline advanced stage cHL has focused on de-escalation strategies, either using PET adapted approaches or with the incorporation of novel agents into treatment protocols. In the RATHL trial, the investigators showed that bleomycin can be safely dropped if a Deauville of 1–3 is achieved after 2 cycles of ABVD with a 3 year PFS of 84.4%, outcomes comparable to 6 cycles of ABVD (3 year PFS of 85.7%) [15]. The ECHELON-1 study, a phase 3 randomized study comparing ABVD versus BV + AVD, showed that BV when incorporated into AVD therapy for all 6 cycles of treatment can eliminate the need for bleomycin with a superior 2 year PFS (82.1% vs. 77.2%) with reduced pulmonary toxicity (1% vs. 3%) but with higher rates of peripheral neuropathy (67% vs. 43%) and neutropenia (58% vs. 45%) compared to ABVD [16]. At 5 years of follow-up, BV + AVD continued to show benefit (PFS 82.2% vs. 75.3%), although there was an increased rate of persistent peripheral neuropathy in the BV + AVD group (19% vs. 9%) [17]. In the recently published updated results, BV + AVD demonstrated OS benefit with a hazard ratio of 0.590 (95% CI 0.396–0.879, *p* = 0.009) and estimated 6 year survival rates of 93.9% and 89.4% for BV + AVD and ABVD, respectively [18]. Secondary cancers were reported in 3.5% of patients receiving BV + AVD (14 solid tumors and 9 hematologic malignancies) and 4.9% in patients receiving ABVD (14 solid tumors and 17 hematologic malignancies). Although fertility was not formally assessed in the study, 114 pregnancies were noted in 82 patients receiving BV + AVD and 81 pregnancies in 61 patients receiving ABVD [18].

Aside from ABVD, BV has also been studied as a bleomycin sparing agent in combination with BEACOPP (bleomycin, etoposide, doxorubicin, cyclophosphamide, vincristine, procarbazine, prednisone)-based regimens in the frontline setting. The German phase 2 randomized safety study (*n* = 52 per group) comparing BreCAPP (brentuximab, etoposide, doxorubicin, cyclophosphamide, procarbazine, prednisone) with BreCADD (brentuximab, etoposide, doxorubicin, cyclophosphamide, dacarbazine, dexamethasone) shown a complete remission rate of 94% and 88% for BreCAPP and BreCADD, respectively [19]. After a median observation of 34 months, 3 year PFS estimates were 89.7% (95% CI 81.0–98.3%) for BreCADD and 90.2% (95% CI 80.9–99.5%) for BreCAPP [20]. The BreCAPP regimen was associated with a significantly higher rate of organ toxic events compared to BreCADD (17% vs. 4%) [19]. Hence, BreCADD was selected for further investigation in an ongoing phase 3 study to compare head-to-head with escalated BEACOPP in the German Hodgkin Study Group HD21 trial (NCT02661503).

Like BV, nivolumab has also been studied in the frontline treatment of advanced stage cHL in an attempt to maintain efficacy and minimize toxicity [21, 22]. The checkmate 205 study investigated the activity of nivolumab across various cHL subsets and treatment regimens across four cohorts of patients. Cohorts A–C were composed of patients with R/R cHL while the patients in cohort D were treatment naïve with stage IIB-IV disease. This cohort (cohort D) initially received nivolumab every 2 weeks for a total of 8 weeks followed by nivolumab + AVD for a total of 3 cycles. After completion of therapy, the CR rate was 67% by central review and 80% by investigator review, while the 21 month PFS was 83% by investigator assessment, on par with the 2 year PFS seen in BV + AVD arm in the ECHELON-1 study. The most common grade 3 toxicity was neutropenia (49%) with 10% of patients having febrile neutropenia. Results of the ongoing Southwest Oncology study (SWOG1826, NCT#03907488), a randomized phase III study comparing BV + AVD versus nivolumab + AVD in newly diagnosed stage III-IV cHL, are eagerly awaited. Table 1 summarizes recently completed and ongoing investigational trials in frontline cHL.

**Table 1:** Recently completed and investigational trials in frontline cHL

Treatment^a^	Early versus advanced	Phase	*N*^b^	ORR% [CR%]	Median follow up (months)	PFS	Publication or NCT#
*All fit patients*							
Pem X 3 -> AVD X 4–6	Early unfavorable	2	30	100 [100]	22.6	100% at 22 months	(13, 14)
Nivo + AVD X 4, Nivo X 2 -> Nivo + AVD X 2 -> AVD X 2	Early unfavorable	2	109 (54, 51)^c^	100, 96 [90, 94]	14, 13	98%, 100% at 12 months	(22)
Deauville 1–3: BV + nivo X 3, ABVD X 2 -> nivo X 3, Deauville > 3: AVD + BV X 4 -> nivo X 3^d^	Early	2	264	–	–	–	NCT03712202
Nivo X 2 -> Nivo + AVD X 3	Advanced	2	51	84 [67]	9.4	92% at 9 months	(21)
BV + AVD versus Nivo + AVD	Advanced	3	987	–	–	–	NCT03907488
*Elderly specific trials*							
BV X 2 -> AVD X 6 -> BV X 4	Advanced	2	48	88 [83]	23	84% at 2 years	(25)
BV	Both	2	26	92 [NR]^e^	59.4	Median PFS: 10.5 months	(23)
BV + dacarbazine	Both	2	19	100 [NR]	58.6	Median PFS: 46.8 months	(23)
BV + bendamustine	Both	2	20	100 [NR]	51.3	Median PFS: 40.3 months	(23)
BV + bendamustine	Advanced	1/2	59	NR [63]^f^	20.6	54% at 2 years	(26)
BV + Nivo	Both	2	20	95 [NR]	19.4	Median PFS: Not reached	(23)
BV + Nivo	Both	2	46	61 [48]	21.2	Median PFS: 21.8 months	(24)

*Pem*, pembrolizumab; *nivo*, nivolumab; *ABVD*, doxorubicin, bleomycin, vinblastine, dacarbazine; *BV*, brentuximab vedotin; *ORR*, overall response rate; *CR*, complete response

^a^Treatment arms separated by commas

^b^Intent to treat number, for trials still recruiting, the target enrollment number is listed

^c^Listed as concurrent, sequential

^d^All patient randomized after ABVD X 2 based on Deauville scores after 2nd cycle, patients starting with bulky disease achieving Deauville 1–3 will not be randomized to the BV + nivo arm

^e^Not reported

^f^Complete response rate reported at 20.6 months of mean follow

### Elderly cHL patients

The elderly cHL patient (age ≥ 60) presents a particularly challenging situation given the difficulty in delivering combination based chemotherapy, especially given the toxicities associated with bleomycin and anthracyclines. There is limited prospective data in this population and no standard approach to treatment currently exists. Novel agents are attractive options to either reduce the amount of chemotherapy in the frontline setting or as part of chemo-free regimens to achieve durable disease control. Several small, chemo-free clinical trials have been conducted in the elderly population. In a multi-arm phase II trial in treatment naïve elderly (age ≥ 60) patients (*n* = 26) which included a BV monotherapy arm [23], an ORR of 92% with a median PFS of 10.5 months was seen, although treatment discontinuation due to adverse events was high at 42% most commonly from peripheral neuropathy (38%). In a small phase II trial (*n* = 46) of elderly patients (> 60 years) who were not candidates for ABVD therapy [24], patients were given doublet therapy with BV (1.8 mg/kg every 21 days) plus nivolumab (3 mg/kg every 21 days). An overall response rate (ORR) of 64% and a complete response rate (CRR) of 52% were seen which did not reach the pre-specified response rate of 80%. The median PFS was 21.8 months. Although these response rates were lower than that seen with ABVD, these results do show the BV + nivolumab combination therapy has activity in the front-line setting and may be a reasonable treatment option for patients who may not tolerate combination chemotherapy. Further, adequately powered randomized studies are still needed to compare the outcomes of starting with BV + nivolumab doublet therapy versus beginning with a monotherapy, either BV or nivolumab, with crossover at the time of progression.

Another approach to reducing toxicity in elderly patients while potentially maintaining efficacy is for sequential delivery of agents to minimize the toxicity associated with the delivery of all the agents of the regimen upfront. In a phase II study, with elderly patients with cHL (age > 60), a sandwich approach was investigated in which BV was administered as a lead in therapy for two cycles followed by 6 cycles of AVD followed by BV for 4 more cycles [25]. With a median age of 69 (range 60–88) and a median Cumulative Illness Rating Scale-Geriatric comorbidity score of 7 (*n* = 48), 52% completed the entire treatment while 77% of patients completed 6 cycles of AVD. In the intent to treat population, the 2 year EFS, PFS, and OS were 80%, 84%, and 93%, respectively. The most common grade 3–4 toxicities were neutropenia (44%), febrile neutropenia and pneumonia (8%), and diarrhea (6%). Peripheral neuropathy was seen in 33% of patients, none of which were higher than grade 2 in severity. Despite the fairly large number of patients unable to complete the full regimen as intended, these results showed that sequential BV and combination chemotherapy may be an alternate approach for treatment delivery, especially for patients who may not tolerate concurrent BV + chemo, but a larger randomized study would be needed to confirm the non-inferiority or superiority of this approach before it can be recommended in routine practice in advanced stage patients. BV added to single agent chemotherapy, notably bendamustine and dacarbazine, has also been studied with median PFS in the range of 40.3–46.8 months [23, 26]. Table 1 summarizes trials of frontline treatment in elderly cHL patients.

#### Key points

For early-stage patients, where CMT is still the standard of care, an important open question is whether novel agents can be incorporated with chemotherapy to spare consolidative RT without compromising PFS. This has been demonstrated in a phase II trial with BV + AVD but larger non-inferiority trials with BV + AVD (or nivolumab + AVD) are needed to change the current standard of care. The alternative PET adapted approach as in NCT03712202 with 2 cycles of ABVD upfront followed by RT free consolidation with novel agents also warrants further evaluation.

For advanced stage patients, BV + AVD remains the standard of care at the present time. However, the ongoing phase III SWOG1826 study will inform us if nivolumab + AVD will become a new bleomycin sparing option in the front-line setting in advanced stage disease. Risk-adapted approaches using immunotherapy or combinations of BV with immunotherapy in either escalation or de-escalation strategies will require further studies.

Chemo-free approaches remain investigational at this point but are an option for elderly or infirm with demonstrated efficacy in the frontline setting. Importantly, chemo-free options do provide a great option to start with for patients with decreased performance status secondary to disease whose status may improve with disease control. These patients may eventually be bridged to a more definitive chemotherapy based regimen, such as BV + AVD, or a milder chemo-containing regimen like BV + dacarbazine. However, chemo-free approaches by themselves will likely ultimately require the recruitment of other biologic therapies with efficacy in the R/R setting into the front-line to get responses on par with that of chemotherapy.

### Relapsed/refractory cHL: salvage therapy after frontline therapy

Approximately 10% of patients with limited stage cHL and 20–30% of advanced stage cHL will progress or relapse after frontline therapy, necessitating salvage treatment in an attempt to achieve long-term remission [27]. Platinum- or gemcitabine-based combination chemotherapy regimens [e.g. ICE (ifosfamide, carboplatin, etoposide), GVD (gemcitabine, vinorelbine, liposomal doxorubicin), or DHAP (dexamethasone, cytarabine, cisplatin)] have historically been the mainstay of second line cHL therapy with ORR and CR rates by PET ranging between 70 and 89% and 54% to 73%, respectively [6, 28–32], with ICE being the most commonly used regimen in the US. There are no randomized trials comparing the efficacy of these regimens and no single regimen has been shown to be superior to another.

Novel agents have been used as a part of salvage therapy regimens to bridge to autologous hematopoietic stem cell transplant (auto-HCT) to eliminate the need for chemotherapy and to increase the response rates to facilitate consolidation with auto-HCT. BV monotherapy as an initial salvage regimen has been studied in a couple of phase II studies, with complete response rates of 27% and 43% [33, 34]. These results show that BV can be used in the 2nd line setting as a chemo-sparing bridge to auto-HCT, although the CR rate in both studies was lower than that seen with traditional salvage chemotherapy. This is also consistent with the low CRR (36%) seen with BV alone in the front-line setting as well [25]. BV has also been studied as a concurrent treatment with salvage chemotherapy regimens including ICE, DHAP, ESHAP, and bendamustine with complete response rates of 69%, 81%, 70%, and 73.6% respectively [35–38]. Response rates of these studies have to be interpreted with caution as many patients in the current era will have received BV as part of their frontline treatment per ECHELON-1.

Nivolumab has also been studied as a 2nd line salvage therapy using a PET adapted approach in a small phase II study (*n* = 39) [39]. Patients received nivolumab 3 mg/kg every 2 weeks for up to 6 cycles. If CR was not achieved, ICE was added to nivolumab (NICE) for 2 more cycles. In patients receiving 6 cycles of nivolumab (*n* = 31) alone, the CR rate was 77%, with 27 patients proceeding to auto-HCT. Pembrolizumab was given concurrently with the standard salvage regimens such as ICE (*n* = 42) and GVD with CR rates of 86.5% and 95%, respectively [40, 41], indicating some of the highest CR rates reported for treatment regimens in the relapsed setting.

Finally, BV + nivolumab as initial salvage therapy was investigated in phase II involving 62 patients [42, 43]. Patients were initially treated with BV on day 1 and nivolumab on day 8 of the first cycle and subsequently given together on day 1 for 3 more cycles followed by response evaluation. After 4 cycles of combination BV + nivolumab therapy, the ORR, and CRR of 85% and 67% with a PFS at 3 years at 77%. The PFS increased to 91% for patients who were successfully salvaged with an auto-HCT. Table 2 summarizes conventional and investigational salvage therapy trials in relapsed cHL.

**Table 2:** Conventional and investigational salvage regimens in relapsed cHL

Treatment	Phase	*N*	ORR%[CR%]	Median follow up	PFS	Publication or NCT#
Conventional Salvage regimens						
ICE	2	65	88 [30]	43 months	58% at 43 months	(28)
GVD	2	91	70 [19]	3.6 years	52% in transplant naïve, 10% in s/p prior transplant	(29)
DHAP	2	102	89 [21]	18 months	NR^a^	(30)
IGEV	2	91	54 [27]	26 months	53% at 3 years	(31)
BEGEV	2	59	83 [75]	5 years	59% at 5 yeas	(32)
Investigational Salvage regimens						
BV + ICE	1/2	42	95 [69]	NR	69% at 1 year	(35)
BV + DHAP	1/2	55	90 [81]	27 months	74% at 2 years	(36)
BV + ESHAP	1/2	66	91 [70]	27 months	71% at 30 months	(37)
BV + bendamustine	1/2	55	92.5 [74]	20.9 months	62.6% at 2 years	(38)
Nivo + ICE	2	39	78 [70]^b^ 100 [86]^c^	10.5 months	79% at 1 year	(39)
Pembro + ICE	2	42	97 [86.5]	27 months	88.2% at 27 months	(40)
Pembro + GVD	2	39	100 [95]	13.5 months^d^	100% at 13.5 months^d^	(41)
BV + nivo	1/2	91	85 [67]	34.3 months	77% at 3 years	(43)

*ICE*, ifosfamide, carboplatin, etoposide; *GVD*, gemcitabine, vinorelbine, doxil; *DHAP*, dexamethasone, high dose cytarabine, cisplatin; *IGEV*, ifosfamide, gemcitabine, vinorelbine; *BEGEV*, bendamustine, gemcitabine, vinorelbine; *BV*, brentuximab vedotin; *ESHAP*, etoposide, methylprednisolone, high dose cytarabine, cisplatin; *Nivo*, nivolumab; *Pembro*, pembrolizumab; *CR*, complete response; *PFS*, progression-free survival

^a^Not reported

^b^After 6 cycles nivo

^c^After 6 cycles of nivo and 2 cycles of Nivo + ICE

^d^Post-transplant follow up

#### Key points

The incorporation of the BV and PD1 blockade into the current salvage treatment paradigm will depend on the prior exposure to these agents. At the present time, in patients with R/R cHL who are not chemo-refractory without high-risk features, standard salvage chemotherapy is still an acceptable approach. However, for patients with primary refractory disease and or with high-risk features, the addition of either BV or PD1 blockade to conventional chemotherapy regimens should be strongly considered given the lower CR rates typically seen with chemotherapy only approaches. For patients with prior BV exposure, our preference would be for a combination of PD1 blockade and salvage chemotherapy given the impressive CR rates seen with these regimens. For BV naïve patients, BV + nivolumab is also attractive given its excellent PFS, outpatient administration, and favorable toxicity profile.

### Post-transplant consolidation

Given the still high number of patients who eventually relapse after auto-HCT, especially in the high-risk patient, there has been considerable interest in extending PFS or even achieving long-term disease remission with time-limited therapy post HCT. The AETHERA study was a global phase 3 trial that investigated if BV maintenance therapy after auto-HCT in high-risk patients (defined as patients having primary refractory disease (failure to achieve complete remission), an initial remission duration of less than 12 months, or extranodal involvement at the start of pre-transplantation salvage chemotherapy), could extend post HCT PFS [44, 45]. A total of 329 patients were randomized to either observation or brentuximab therapy given every 3 weeks for up to 16 cycles. At 5 years of follow-up, the 5 year PFS was 59% in BV arm versus 41% in the placebo (HR 0.521, 95% CI 0.379–0.717). As expected, peripheral neuropathy was higher in the BV arm (*n* = 76 BV, *n* = 19 placebo), but 90% of patients reported resolution of their peripheral neuropathy at 5 years of follow-up. Post hoc analyses showed that patients with greater than or equal to 2 risk factors (initial remission duration of less than 12 months or primary refractory disease, best response of either PR or stable disease to most recent salvage, extranodal disease at pre-auto-HCT relapse, B symptoms at pre-transplantation relapse, or greater than or equal to 2 prior salvage therapies) derived a higher treatment benefit. This study led to the current standard of care of BV consolidation post HCT relapse in patients with high-risk cHL.

Due to the high rate of toxicity, most notably peripheral neuropathy with maintenance BV in the clinical trial setting, a recent analysis presented at the ASCO 2022 conference investigated the cumulative dose response relationship of BV in the post auto-HCT maintenance setting [46]. The authors found that only 14% of patients can complete the full course of BV in the real-world setting and showed that only 51–75% of the total BV dose is necessary to attain the full PFS benefit of BV maintenance therapy. Thus, the risks and benefits of BV therapy should be continuously weighed while the patient is receiving maintenance BV, especially at the first sign of progression of peripheral neuropathy.

The incorporation of nivolumab into BV maintenance has been investigated in a phase II study involving 59 patients with preliminary results presented at ASH 2020 [47]. Patients received BV (1.8 mg/kg) and nivolumab every 21 days for up to a total of 8 cycles. The primary endpoint was 18 month PFS. Forty-nine percent of patients completed all 8 cycles, with 76% of patients completing 8 cycles of one drug. At a median follow-up of 15.7 months, the estimated 18 month PFS was 95% (92% and 89% in patients with ≥ 2 and ≥ 3 risk factors, respectively). These encouraging initial results indicate the high potential for combined BV + nivolumab time limited therapy in producing a higher rate of sustained remissions in high-risk post HCT patients. Pembrolizumab has been studied in the post-HCT consolidation setting as monotherapy with promising results [48]. Ninety percent of patients enrolled in this study had high-risk features with 20% having prior BV exposure. Pembrolizumab was given for 8 cycles starting 21 days after HCT. At 18 months, the PFS was 82%, with OS of 100%. Longer follow-up of these post HCT PD1 blockade studies will be needed to see if a greater number of durable responses can be achieved with this approach compared to BV consolidation.

#### Key points

In light of the increasing use of BV in the post-ECHELON-1 era and the excellent results with PD1 blockade in the post-transplant setting, the best approach to maintenance therapy in this setting, whether it be the use of BV, PD1 blockade, or combined CD30/PD1 blockade remains an important question. Randomized studies and larger outcomes based studies in the post-ECHELON-1 era will be invaluable in addressing this question.

### Relapsed/refractory cHL: Following auto-HCT

The introduction of anti-CD30 and anti-PD1 therapies into the treatment landscape for the cHL patient with relapsed disease following auto-HCT failure or for the patient unable to undergo auto-HCT has significantly improved survival compared to the historically poor outcomes in this patient population. The pivotal phase II study of BV in R/R cHL patients (*n* = 102) in which BV (1.8 mg/kg) was given once every 3 weeks for up to 16 cycles, produced an ORR and CR rate of 75% and 34%, respectively with a median PFS of 5.6 months, ultimately leading to its approval in cHL after failure of 2 prior lines of treatment [49]. The rate of peripheral neuropathy was high at 42% which frequently limited the duration patients were able to continue therapy. Updated results at the 5 year mark showed an estimated PFS and OS of 22% and 41%, respectively, which was significantly longer in those who achieved CR [50].

Phase II trials of PD1 blockade in R/R cHL have been evaluated for both nivolumab and pembrolizumab. In the checkmate 205 study [21], patients who relapsed after auto-HCT were given nivolumab every 2 weeks at 3 mg/kg until disease progression or unacceptable toxicity. Patients (*n* = 243) were grouped into three cohorts based on their treatment history: no prior BV (cohort A, *n* = 63), maintenance BV after auto-HCT (cohort B, *n* = 80), and BV received before or after auto-HCT with the intent of disease control (cohort C, *n* = 100). While the ORR was similar across the 3 cohorts, the CR rate was higher in cohort A (29%) compared to cohorts B (13%) and C (16%). The median PFS of 14.7 months. These results led to the accelerated approval of nivolumab for patients who relapsed or progressed after auto-HCT and post-transplantation BV. In the pivotal Keynote-087 study, patients who either relapsed after auto-HCT plus BV (cohort 1), had chemo-refractory disease and unable to move on to auto-HCT (cohort 2), or relapsed after auto-HCT (cohort 3), received pembrolizumab 200 mg every 3 weeks. The ORR and CR rate was 69.0% and 22.4%, with ORR similar across the three cohorts. At 2 years of follow-up, the median duration of response was 16.5 months, with a longer duration of response in cohort 1 (22.1 months) and cohort 3 (24.4 months) compared to cohort 2 (11.1 months) [51]. These results indicate significant activity of pembrolizumab in heavily treated patients with cHL.

These overall positive results and longer durability of responses with PD1 blockade in phase II trials of R/R cHL naturally led to the question of which modality of therapy, either PD1 blockade or anti-CD30 therapy, would lead to better outcomes for patients who failed at least one line of treatment in cHL. This was formally tested in the Keynote-204 study in which patients (*n* = 304) were randomized to receive either pembrolizumab 200 mg IV Q3W or BV 1.8 mg/kg Q3W [52]. Patients who had either relapsed after auto-HCT (37% of patients in both groups) or were not candidates for auto-HCT (63% of patients in both groups) were included in addition to patients who had responded to prior BV (3% in pembrolizumab group, 7% in BV group). The primary endpoint was PFS by independent review. After a median follow-up time of 25.7 months, the median PFS was 13.2 months (95% CI 10.9–19.4) for pembrolizumab versus 8.3 months (95% CI 5.7–8.8) for BV (*p* = 0.00027). The most common grade 3 or higher treatment related adverse events in the pembrolizumab versus BV groups were pneumonitis (4% vs. 1%), neutropenia (2% vs. 7%), decreased neutrophil count (1% vs. 5%), and peripheral neuropathy (1% vs. 3%), with the overall frequency of serious adverse events being 16% versus 11%. Patients discontinuing due to adverse events was higher in the BV arm (13.5% in pembrolizumab group vs. 19% in BV group) while disease progression while on therapy was lower with pembrolizumab compared to BV (39.1% vs. 49.3%), indicating likely a combination of better therapy tolerance as well better therapeutic efficacy as reasons for the superior outcome of pembrolizumab versus BV. These results led to the FDA to extend the indication for pembrolizumab to all adult patients with R/R cHL.

Further improvement in the responses and outcomes of PD1 blockade in R/R cHL using combination approaches with other investigational agents is an active area of research (Table 3). Nivolumab has been combined with ipilimumab in a phase Ib study (*n* = 31) showing an ORR and CRR of 74% and 23%, respectively [53]. The addition of BV to nivolumab + ipilimumab has also been investigated in a phase I study showing an ORR and CRR of 82% and 73% [54]. Preliminary results of a small phase II study (*n* = 11) combining the Bruton’s Tyrosine Kinase (BTK) inhibitor ibrutinib with nivolumab in patients with R/R cHL which included patients with prior nivolumab exposure showed an ORR and CRR of 60% and 40% with median PFS not yet reached at a median follow up time of 9.5 months [55]. The Keynote-145 study is an ongoing phase 1b/2 study combining acalabrutinib with pembrolizumab in various hematologic malignancies, including cHL with a target enrollment of 161 patients (NCT02362035) Use of other cellular therapy approaches (see below) in addition to PD1 blockade may also yield deeper and more durable responses, but these cellular approaches are still in the early stages of development.

**Table 3:** Investigational combinations and novel therapies in relapsed cHL

Treatment	Phase	Total *n*	ORR% [CR%]	Median follow up	Median PFS	Publication or NCT#
Nivo + ipilimumab	1b	31	74 [23]	NP^a^	NR	(53)
Nivo + ipilimumab	1b	21	76 [24]	NP	NR	(53)
Nivo + ipilimumab + BV	1/2	64	82 [57]	1.7 years	NR	(54)
Nivo + ibrutinib	2	10	66 [44]	9.5 months	NR	(55)
Pembro + acalabrutinib	1b/2	161^c^	–	–	–	NCT0236035
Cami-T	2	117	70.1 [33.3]	10.7 months	9.1 months	(63)
Pembro + favezelimab	1/2	33	31 [7]	16.5 months	9 months	(66)
CD30 CART	2	41	72 [59]	17.8 months	14.8 months^d^	(69)
CD30 CART	2	97^c^	–	–	–	NCT04268706
Anti-EBV directed cell therapy	2	25	61.9 [52.4]	NP	NP^e^	(71)
Anti-EBV/DN TGF beta cell therapy	1	8	57.1 [28.6]	NP	NP	(72)
Anti-EBV/CD30 dual CART	1	18^c^	–	^−^	^−^	NCT01192464

*Nivo*, Nivolumab; *BV*, Brentuximab vedotin; *Pembro*, pembrolizumab; *CART*, Chimeric antigen receptor T-cells; *EBV*, Epstein-Barr Virus; *DN TGF*, Dominant Negative Tumor growth factor; *CR*, complete response; *PFS*, progression-free survival

^a^Not presented

^b^Not reached

^c^Target enrollment

^d^For patients who had a CR

^e^2-year EFS for all patients including other lymphomas with active disease was 50%

#### Key points

With the results of the Keynote-204 study, pembrolizumab is now a preferred treatment for patients who relapsed after auto-HCT and who have not previously progressed on immunotherapy on a clinical trial. The addition of BV to immunotherapy in patients who have not previously progressed on BV is still investigational while BV monotherapy is still a potential option after immunotherapy failure.

### Options for double refractory cHL patients (refractory to both BV and CPI)

After the exhaustion of anti-PD1 and anti-CD30 therapies, options for the heavily pretreated cHL patients are limited and largely palliative in nature. Use of chemotherapy regimens with activity in NHL, such as bendamustine, gemcitabine + oxaliplatin (Gem-Ox), or bendamustine + carboplatin + etoposide (TEC) has shown activity in multiply relapsed cHL patients [56–58]. Other non-chemotherapeutic targeted options such as lenalidomide and everolimus have also shown activity in R/R cHL with ORR of 30% and 46%, respectively, with a median PFS of 8 months for each [59, 60]. In this section, we discuss several promising investigational therapies within this patient population, including camidanlumab tesirine (Cami-T), anti-lymphocyte activated gene 3 (LAG3) therapy, chimeric antigen receptor T (CART) therapy, Epstein-Barr virus (EBV) directed anti-cHL cytotoxic cellular therapy, and allogeneic HCT (Table 3).

### Small molecule inhibitors

Camidanlumab Tesirine (Cami-T) is an antibody drug conjugate composed of an antibody directed against CD25 (IL-2R alpha) conjugated to a pyrrolobenzodiazepine (PBD) dimer toxin [61]. Upon receptor binding, the PBD toxin is internalized and results in DNA crosslinking and cell death. In addition to CD25 being expressed on HL cells, there may also be an immune stimulatory effect by depletion of CD25-expressing T-regulatory cells within the HL microenvironment. A large, phase I dose escalation study in R/R cHL (*n* = 60) showed it to be relatively safe with the minimum tolerated dose not reached [62]. Notably, 2 patients (3.3%) did develop Guillain–Barre syndrome (GBS) as an immune related side effect. with an ORR and CRR of 69.1% and 43.6%, respectively. Updated results of the ongoing pivotal phase 2 study (*n* = 115) of Cami-T presented at the 2022 European Hematology Association (EHA) conference showed an ORR and CRR of 70.1% and 33.3%, respectively, with a median duration of response of 13.7 months in a heavily pretreated (median number of prior treatments = 6) cohort of patients with R/R cHL [63]. GBS was again seen, this time in 6.8% of patients, consistent with the phase I data, although the authors indicated that the GBS symptoms could be mitigated by medical intervention. These promising results indicate that Cami-T may be a potential therapy for multiply relapsed cHL [61]. However, there needs to be a detailed discussion regarding the risk of GBS prior to instituting the therapy. The treating providers need to be vigilant regarding this adverse event and request expert neurological evaluation immediately once GBS/radiculopathy is suspected, as the start of therapeutic intervention (high dose steroids ± plasma exchange) immediately following the diagnosis of this complication are key to rapid resolution of this toxicity..

Lymphocyte activated gene 3 (LAG3) is surface protein expressed on the surface of conventional T-cells and T-reg cells and results in inhibition of T-cell activation as well as cytokine and granzyme secretion [64]. Just like PDL1, LAG3 is nearly always expressed within the tumor microenvironment of cHL [65]. Favezelimab is a humanized IgG4 LAG3 inhibitor studied in an open label, multi-cohort phase 1/2 study combining favezelimab with pembrolizumab is currently ongoing in hematological malignancies (NCT03598608), with cohort 2 composed of patients with cHL who had either relapsed or were ineligible for auto-HCT and who were refractory to prior anti-PD1 blockade. Preliminary results of this combination showed an ORR and CRR of 31% and 7% with a median PFS of 9 months (*n* = 33) [66]. These results suggest likely independent activity of favezelimab when combined with PD1 blockade in the setting of prior PD1 blockade. The future of this combination will likely reside in its benefit for PD1 blockade-naïve patients, especially given the favorable recent results of pembrolizumab from Keynote-204.

### Cellular therapies

Perhaps no other therapy has garnered as much excitement in lymphoma treatment over the past decade as CART cells [67]. Given the near universal bright expression of CD30 on the Reed Sternberg cells of cHL and its validation as a therapeutic target [68], it is not surprising that anti-CD30 CAR-Ts (CD30.CART) became the first CART investigated in R/R cHL. In recent phase I/II studies conducted at two centers to evaluate the efficacy of a CD30.CART, ORR, and CR rate in 32 patients with active disease at the time of infusion was 72% and 59%, respectively [69]. However, most responses were not durable, as the 1 year PFS was 36%. Interestingly, CD30 expression was still retained on lymphoma cells at the time of relapse, suggesting that other mechanisms aside from antigen loss, such as the immunosuppressive cHL tumor microenvironment, may be playing a role in CD30.CART cell resistance. A larger, multicenter phase II study (CHARIOT) with a target enrollment of 97 patients is currently ongoing with an estimated completion year of 2025 (NCT04268706).

Approximately 40% of patients with cHL have an expression of either the EBV antigens latent membrane protein 1 (LMP1) or latent membrane protein 2 (LMP2) (type II EBV latency pattern) and thus are attractive, specific targets for cellular therapy but are generally considered to be weakly immunogenic in this context [70]. Patients with EBV + cHL cells generally harbor low levels of these antigen specific T-cells but can be expanded ex vivo with and without further engineering and ultimately reintroduced as a form of EBV directed anti-cHL cytotoxic cellular therapy. In a trial treating a heterogenous mix of patients with several different EBV associated lymphomas [71], including HL (*n* = 25), who either had active disease or were at high risk of relapse, showed an ORR of 61.9% (13 of 21) and CRR of 52.4% (11 of 21). A subsequent study built on this therapeutic platform further by expression of a dominant-negative TGF-beta receptor type 2 within the EBV specific cytotoxic cells, leading to a response in 4 out of 7 patients, 2 of which were complete responses [72]. A phase I trial investigating a dual specificity cellular therapy using EBV specific cytotoxic cells engineered to express an anti-CD30 CAR is currently underway (NCT01192464).

### Allogeneic HCT

At the present time with available treatment options, patients who progress following auto-HCT typically do not achieve long-term disease control without the use of allo-HCT. Several retrospective series have shown the survival advantage of patients undergoing allogeneic HCT (allo-HCT) in patients with R/R cHL after failure of auto-HCT and remains a potentially curative option in heavily pretreated patients [73–75]. Reduced-intensity conditioning has significantly improved the outcomes compared to traditional myeloablative regimens and is the conditioning approach of choice for cHL [76]. However, as in the case of NHL, in the era of BV, PD1 blockade and clinical trial options, the role of allo-HCT after the failure of auto-HCT may not be as clear as it was in the past when other post auto-HCT options did not exist [77]. Regardless, all patients fit to undergo an allo-HCT should be at least considered for this approach in the post-HCT setting. A nice algorithm to follow after failure of auto-HCT in the ear of novel agents has been previously published [77]. Patients with primary refractoriness to the first line and salvage chemotherapy regimens who are able to achieve a response with novel agents should be strongly considered for allo-HCT. A common question that arises is whether or not a patient who achieves good disease control, and not necessarily a CR, to either brentuximab or PD1 blockade should proceed to an allo-HCT [73], as data suggests that pre-transplant Deauville scores may have less prognostic relevance in cHL as it does in DLBCL [78–80] This is typically the favored approach in the case of the younger patient with a significantly longer life expectancy who would be expected to eventually exhaust available treatment options.

Another important related question is if either pre-allo-HCT (bridging) or post-allo-HCT (consolidative or salvage) therapies have any specific beneficial impact in this setting. In the pivotal phase II trial of brentuximab therapy in the post-auto-HCT patient, 4 patients in CR were consolidated with allo-HCT, all of which remained in CR. However, as 9 patients who were in CR also remained in CR without consolidative allo-HCT, the benefit of consolidative allo-HCT in the patient who has been in a prolonged CR to brentuximab is unclear.

The benefits and risks of both pre and post allo-HCT PD1 blockade has been studied in greater detail. In the checkmate 205 study, 44 patients eventually were able to proceed to allo-HCT [81]. In this patient subset, the 6 month cumulative incidence of transplant related mortality (TRM) was 13%, with 6-month estimated PFS of 82%. A multi-center retrospective analysis of 39 patients with lymphomas, 31 of which were cHL, treated with PD1 blockade before undergoing allo-HCT, the cumulative incidence of grade 2–4 and grade 3–4 GVDH was 44% and 23%, respectively, with four treatment related deaths. In a smaller series of 13 patients with R/R cHL who received PD1 blockade as bridging therapy to allo-HCT [82], all patients had a CR post-allo-HCT. The PFS and OS at 57.4 months was 75.5% and 90.9%. Thirty-eight percent of patients developed acute GVHD, with only one patient dying due to grade 3 GVHD involving the liver. Taken together, these results suggest that there is likely a greater risk of GVHD with the use of PD1 blockade in the pre-allo-HCT setting, but this risk may be offset by the benefit of long-term remission with allo-HCT after PD1 blockade.

Another important scenario to consider is the potential effect of re-activating an allograft in the setting of post allo-HCT progression with PD1 blockade. In a series of 20 patients with cHL with relapse after undergoing an allo-HCT who were then treated with nivolumab [83], 6 patients (30%) had GVHD after nivolumab, 2 of which died. All patients with GVHD after nivolumab had GVHD prior to their course. The ORR was 95% with a 1 year PFS and OS of 58.2% and 78.7%. This high ORR compares favorably to the ORR of 69% seen in the Checkmate-205 study, even in the context of the more heavily pre-treated patients in this course. As these patients were PD1 blockade naïve, this scenario is likely an uncommon occurrence as most patients would receive PD1 blockade at some point prior to allo-HCT. However, it does raise the question of whether patients in the post-allo HCT setting should receive consolidative PD1 blockade particularly after immunosuppression is withdrawn to achieve a higher rate of durable responses. A strong case can be made for this in the patient in which no acute GVHD occurred and who had a good response to PD1 blockade at some point prior to allo-HCT.

#### Key points

Patients who are double refractory should be encouraged to enroll in clinical trials as there is no standard of care in this patient population. Cami-T is a promising therapy in this setting that may be soon approved, although there is a risk of GBS as previously discussed. Although cellular therapy has not been approved for R/R cHL, CAR30.CART cells are currently being investigated in a phase II study. Allo-HCT should be strongly considered in this setting for eligible patients and remains a potentially curative therapy even in the age of novel agents and immunotherapy.

## Concluding perspectives on the future of cHL treatment

While CMT has remained the standard of care for early-stage cHL patients for over a decade, the incorporation of BV into the frontline chemotherapy (BV + AVD) for advanced stage cHL has changed the therapeutic landscape in cHL patients. Given the impressive results of PD1 blockade in multiply relapsed patients, as first salvage, and as a post-HCT consolidation approach, there is a good chance that these improved outcomes will be carried over to the frontline setting to define a new standard of care. Results of SWOG1826 will require time to mature given the already good PFS results with BV + AVD therapy. Biomarker based stratification of patients has remained elusive in cHL but should still be actively pursued as these may be more important than ever before as new targeted therapies are introduced. The treatment approaches in the salvage setting will likely be dictated by prior therapies. PD1 blockade will be an important second line therapy in patients who progressed on prior BV and vice versa. The combinatorial approach (novel agents + chemotherapy) should be strongly considered to provide the highest chance for achieving deeper remissions. Finally, there has been much excitement moving past BV and PD1 blockade into newer cellular and immunotherapy approaches given the rich inflammatory cHL microenvironment.

In conclusion, despite achieving remarkable improvements over the past 60 years in the outcomes of patients with cHL, there remains plenty of room for further improvement in the treatment of not only multiply relapsed cHL but also the treatment naïve cHL patients. There are many exciting avenues of ongoing research in cHL that will continue to move the needle in the right direction and improve the outcomes of these patients.

## Data Availability

Not applicable.

## Abbreviations

ABVD: Doxorubicin, bleomycin, vinblastine, dacarbazine
BV: Brentuximab vedotin
BTK: Bruton’s tyrosine kinase
CAMI-T: Camidanlumab tesirine
CART: Chimeric antigen receptor T-cell
CD: Cluster of differentiation
CMR: Complete metabolic response
CMT: Combined modality therapy
CPI: Checkpoint inhibitors
CRR: Complete response rate
cHL: Classical Hodgkin’s lymphoma
DHAP: Dexamethasone, cytarabine, cisplatin
EBV: Epstein-Barr virus
EFS: Event free survival
EHA: European Hematology Association
FDA: Food and Drug Administration
GBS: Guillain–Barre syndrome
GHSG: German Hodgkin’s Study Group
GVD: Gemcitabine, vinorelbine, liposomal doxorubicin
HCT: Hematopoietic stem cell transplant
ICE: Ifosfamide, carboplatin, etoposide
LAG3: Lymphocyte activated gene 3
NHL: Non-Hodgkin’s lymphoma
NLPHL: Nodular lymphocyte predominant Hodgkin’s lymphoma
ORR: Overall response rate
OS: Overall survival
PBD: Pyrrolobenzodiazepine
PET: Positive emission tomography
PD1: Program death receptor 1
PFS: Progression-free survival
R/R: Relapsed/refractory
RT: Radiotherapy
TGF: Tumor growth factor
TRM: Transplant related mortality
